# Multicompartment Nanostructures as Templates for Multimetallic Hybrid Materials

**DOI:** 10.1002/smsc.202300071

**Published:** 2023-07-21

**Authors:** Stefanie Tjaberings, Markus Heidelmann, Steffen Franzka, André H. Gröschel

**Affiliations:** ^1^ Institute for Physical Chemistry and Center for Soft Nanoscience (SoN) University of Münster Corrensstraße 28-30 48149 Münster Germany; ^2^ Interdisciplinary Center for Analytics on the Nanoscale (ICAN) University of Duisburg-Essen Carl-Benz-Str. 199 47057 Duisburg Germany; ^3^ Macromolecular Chemistry and Bavarian Center for Battery Technology (BayBatt) University of Bayreuth Weiherstraße 26 95448 Bayreuth Germany

**Keywords:** ABC triblock terpolymers, hybrid materials, morphologies, multimetallic nanostructures, templates

## Abstract

ABC triblock terpolymers are promising soft templates for organic/inorganic hybrids because of their ability to form nanostructures with complex shapes, multiple compartments, and precisely localized chemistry. Exemplified on multicompartment nanofibers (MCNFs) of triblock terpolymers, it is demonstrated that microdomains can be selectively loaded, thereby giving access to nanoscale multimetallic hybrid materials. MCNFs with micrometer length, homogenous diameter (90 nm), and a helix‐on‐cylinder morphology are formed from polystyrene‐*block*‐polybutadiene‐*block*‐poly(*tert*‐butyl methacrylate) (PS‐*b*‐PB‐*b*‐PT). After postmodification (cross‐linking/hydrolysis), selective loading with FeCl_3_, PdCl_2_, H_2_PtCl_6_, AgNO_3_, CuCl_2_, or ZnCl_2_ leads to a variety of hybrid MCNFs analyzed by transmission electron microscopy, scanning transmission electron microscopy, electron tomography, energy‐dispersive X‐ray spectroscopy, and atomic force microscopy. Mild sulfonation of the PS shell to polystyrene sulfonate renders the MNCFs water‐dispersible and allows the formation of mixed‐bimetallic Pt/Pd/Pt@MCNFs and trimetallic Pt/Pd/Ag@MCNFs. It is demonstrated that the order of loading is key to successfully create multimetallic nanostructures. These and other structures can become useful for energy applications as well as in photo‐ and electrocatalysis.

## Graphical abstract

Multicompartment nanostructures of ABC triblock terpolymers are used for the template‐directed synthesis of mono‐, bi‐, and trimetallic nanohybrids. Polystyrene‐*block*‐polybutadiene‐*block*‐poly(*tert*‐butyl methacrylate) (PS‐*b*‐PB‐*b*‐PT) is converted to multicompartment nanofibers with PT core, PB double helix, and PS corona, and employed soft and selective template for various metal precursors (e.g., Fe, Ag, Cu, Pt, Pd, Zn) to form complex hybrid nanostructures.

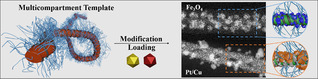

## 1 Introduction

The template‐supported formation of nanoscale metal/metal oxide shapes is an important concept in materials science and nanotechnology. In nature, examples like siliceous sponges, diatom algae (frustules), or seashells form their rigid skeleton through biomineralization, which is assisted by soft biopolymer templates that guide the mineralization process into characteristic and intricate architectures.^[^ ^1^, ^2^, ^3^, ^4^ ^]^ Growing inorganic materials in soft scaffolds thereby gives access to shapes and forms that are otherwise difficult to realize. Creating highly defined shapes of inorganic nanoparticles (NPs) has a long tradition in materials science using either a top‐down strategy to carve out NPs from larger objects or a bottom‐up strategy that relies on the reduction of metal salts in the presence of stabilizing or capping agents.^[^ ^5^, ^6^, ^7^ ^]^ These directing agents control NP growth and typically include low molecular surfactants,^[^ ^8^, ^9^ ^]^ ionic (bio)polymers,^[^ ^10^, ^11^ ^]^ polymer brushes,^[^ ^12^ ^]^ and block copolymer (BCP) nanostructures.^[^ ^13^ ^]^ While spherical NPs^[^ ^14^, ^15^, ^16^ ^]^ are comparably straightforward to produce, more sophisticated methods are required to stabilize interfaces of increasing anisotropy, where rods,^[^ ^17^ ^]^ rings,^[^ ^18^ ^]^ and platelets^[^ ^19^ ^]^ have been successfully prepared as well as cubes,^[^ ^20^, ^21^ ^]^ triangles,^[^ ^22^ ^]^ and octapods.^[^ ^23^ ^]^ In order to create more complex 3D forms, BCPs are particularly versatile templates for energy conversion and photonics.^[^ ^24^, ^25^ ^]^ For instance, self‐assembled cylindrical, gyroidal, and lamellar microdomains in bulk morphologies as well as nanoporous films^[^ ^26^, ^27^ ^]^ of polystyrene‐*block*‐poly(4‐vinylpyridine) (PS‐*b*‐P4VP) or poly(ethylene oxide)‐*block*‐poly(4‐vinylpyridine) (PEO‐*b*‐P4VP) were employed for the synthesis of well‐defined silica or Pd/silica nanostructures.^[^ ^28^ ^]^ Amphiphilic or bis‐hydrophilic templates were utilized to coordinate and direct the growth of magnetic NPs^[^ ^29^, ^30^ ^]^ or 1D necklace‐like structures consisting of nanodiscs of semiconducting, magnetic, or ferroelectric nanocrystals.^[^ ^31^ ^]^

Aside from monometallic NPs, also bi‐ and multimetallic NPs are pursued either in form of alloys or multicompartment NPs.^[^ ^32^ ^]^ Especially for polyelemental NPs,^[^ ^33^ ^]^ the formation process is not fully understood as recently investigated on Pd/Sn‐based alloys with mixed‐composition of up to seven elements.^[^ ^34^ ^]^ Multicompartment NPs on the other hand exhibit clearly defined domains and are produced by stepwise reduction, where thermodynamic immiscibility and phase‐segregation have to be considered.^[^ ^35^ ^]^ The seed‐mediated solution‐phase technique thereby gives access to core–shell NPs, e.g., a Au core is formed in a first reduction step followed by the formation of a Ag shell toward bimetallic Ag@Au core–shell NPs.^[^ ^36^, ^37^, ^38^ ^]^ Also, the growth of a Pd shell on a Au core was reported displaying synergy for catalysis.^[^ ^39^ ^]^ Such multicompartment NPs could also exhibit the potential for commercially viable hydrogen fuel cells with high turnover frequencies.^[^ ^40^ ^]^

To form multimetallic NPs with more complex shapes with block copolymer nanostructures, polymers need to have three domains, where at least two are specifically reserved for loading. In this regard, ABC triblock terpolymers^[^ ^41^, ^42^, ^43^ ^]^ are versatile templates to accommodate multiple inorganic materials.^[^ ^44^ ^]^ They consist of three chemically different blocks that form nanostructures with three nanodomains through self‐assembly in solution,^[^ ^45^ ^]^ in confinement,^[^ ^46^, ^47^, ^48^ ^]^ or in bulk.^[^ ^49^, ^50^, ^51^, ^52^, ^53^, ^54^ ^]^ These domains have dimensions in the range of 10–100 nm and can be designed with specific geometry controlled by block length ratio. There are several examples in bulk, where one is mesoporous NbN superconductors with gyroid morphology could, for instance, be generated from PI‐*b*‐PS‐*b*‐PEO double gyroid bulk films (PI = polyisoprene).^[^ ^55^ ^]^ There, the PEO gyroid was selectively loaded by co‐casting with Nb_2_O_5_ and the NbN gyroid obtained after calcination and nitriding steps. PS‐*b*‐P4VP‐*b*‐PEO triblock terpolymers also demonstrated intriguing pattern formation on surfaces utilized for templating of Au‐ and Ag‐NP pattern.^[^ ^56^ ^]^ Next to the salt accommodating PEO, the vinyl pyridine units are likewise able to coordinate metal precursor as demonstrated on self‐assembled PS‐*b*‐P2VP‐*b*‐PEO core–shell–corona micelles that allowed creating bimetallic spherical and cylindrical Pt/Au NPs.^[^ ^57^ ^]^ There, the Pt precursor salt was selective to the P2VP domain, while the Au precursor accumulated with the PEO domain. Precise molecular control over domain dimension is provided by ABC terpolymer brushes as recently demonstrated for spherical^[^ ^58^ ^]^ and cylindrical^[^ ^59^ ^]^ core–shell–corona brushes. Such core–shell–corona architectures are the simplest form of compartmentalization, while there is a much wider range of multicompartment nanostructures that have, so far, not been investigated as templates for hybrid materials, e.g., spheres‐on‐sphere, spheres‐on‐bilayers, striped and perforated discs, as well as perforated vesicles, just to name a few.^[^ ^60^ ^]^ We recently utilized one of these multicompartment nanostructures and reported on the selective formation of hybrid Pt double helices in multicompartment nanofibers (MCNFs) of polystyrene‐*block*‐polybutadiene‐*block*‐poly(*tert*‐butyl methacrylate) (PS‐*b*‐PB‐*b*‐PT).^[^ ^61^ ^]^

Here, we expand on this concept and show that the PB domain can accommodate other metal and metal oxide materials to generate a variety of hybrid double helixes. The MCNF template was further modified by hydrolysis of the PT core to PMAA, to generate bimetallic hybrid nanostructures through selective loading of PMMA core and PB double helix. Finally, the PS corona was transformed to poly(styrene sulfonate) to provide a third coordination site paving the way to trimetallic hybrids. With the aid of element specific microscopy analysis, we provide general guidelines for successful multimetal loading in ABC triblock terpolymer templates.

## 2 Results and Discussions

### 2.1 Template Formation

**Scheme 1:**
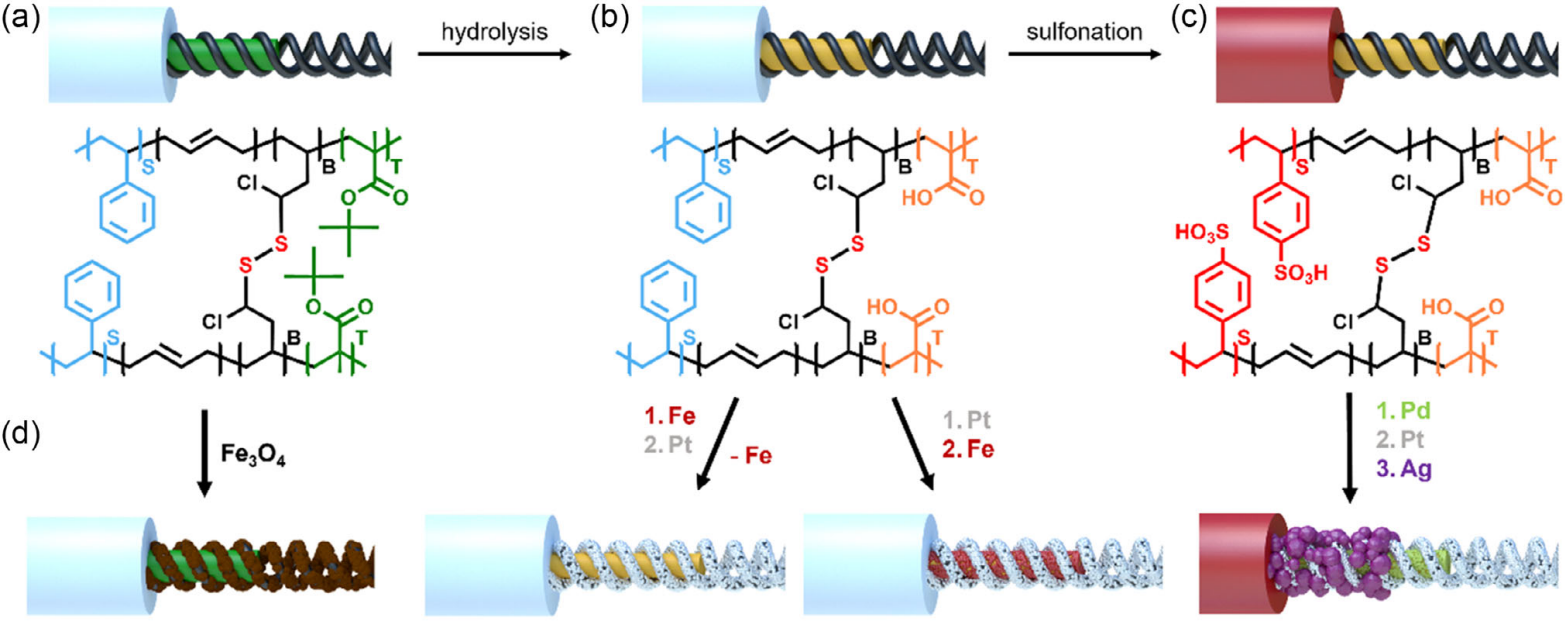
Modification of SBT MCNFs. a) MCNFs with PS corona (gray), PB double helix cross‐linked with S_2_Cl_2_ (black), and PT core (green), as well as chemical structure of the SBT triblock terpolymer (≈ 80% of 1,2‐PB units). b) Hydrolysis of the PT core to PMAA (orange) with TFA. c) Mild sulfonation of PS to a PSS (red). d) Loading examples of the MCNFs at each stage of modification.

The S_66_B_11_T_23_^85^ triblock terpolymer for this study was synthesized via anionic polymerization as reported previously (subscripts denote the weight fraction, wt%, and the superscript the overall molecular weight, *M*_*n*_, in kg mol^−1^).^[^ ^60^ ^]^ The SBT terpolymer forms a multicompartment template that features microdomains with different functional groups and therefore opens the possibility for selective loading with metal precursors as outlined in **Scheme** **1**. The first step is the preparation of a SBT cylinder morphology in bulk cast from CHCl_3_ (*c* = 100 g L^−1^) (Figure S1a,b, Supporting Information). Then, the PB microdomain is cross‐linked with sulfur monochloride (S_2_Cl_2_) stabilizing the PB double helix (diameter 35 nm, pitch 25 nm, thickness 12 nm) (Scheme 1a, Figure S1c,d, Supporting Information).^[^ ^61^ ^]^ The disulfide bridges later serve as coordination sites for salts and allow the redispersion of the helix‐on‐cylinder morphology in dichloromethane (DCM). There, we obtain MCNFs with a length of up to 20 μm and a diameter of 90 nm including PS corona (28 nm), PT core (15 nm), and a PB helix on the core surface. For bimetallic loading, we hydrolyzed the PT core with trifluoroacetic acid (TFA) to poly(methacrylic acid) (PMAA) serving as a second coordination site (Scheme 1b, Figure S1e,f, Supporting Information). Finally, for trimetallic loading, mild sulfonation of the PS corona to poly(styrene sulfonic acid) (PSS) was performed with an acetyl sulfate solution providing the third coordination site, giving access to all three compartments of the MCNFs therein (Scheme 1c). The MCNFs can be loaded with appropriate precursor salt at any stage of this modification process, thereby opening the way to mono‐, bi‐, or trimetallic loading (Scheme 1d).

### 2.2 Monometallic Loading

**Figure 1:**
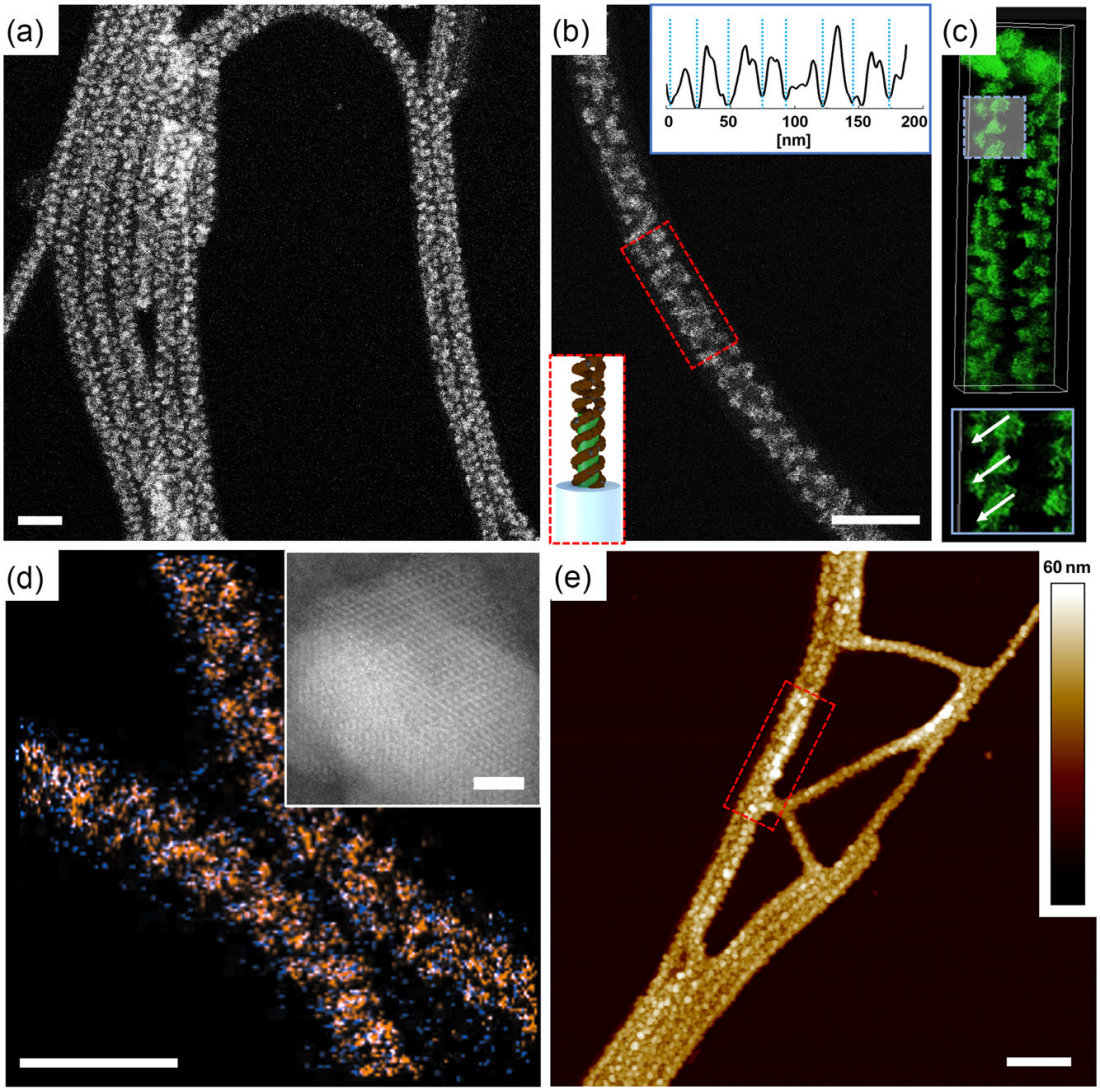
SBT MCNFs loaded with Fe_3_O_4_ NPs. a) STEM dark‐field image of Fe_3_O_4_@MCNFs. b) Close‐up of nanofiber with scheme and grayscale analysis of the marked areas. c) 3D reconstruction of helical arranged Fe_3_O_4_ NPs within the PB compartment. d) Overlap of EDX spectra of iron (orange) and oxygen (blue). The inset demonstrates the crystal structure of the Fe_3_O_4_ NPs. e) AFM height image of loaded Fe_3_O_4_@MCNFs. (Scale bars: (a–c) 100 nm, (d) 400 nm and inset 1 nm, and (e) 200 nm).

As a first example, we formed Fe_3_O_4_ NPs within the MCNFs in a similar manner as before Pt in previous work (**Figure** **1**). For that, the SBT MCNFs were transferred to DMF, infiltrated with equimolar amounts of FeCl_2_ and FeCl_3_, and reduced to Fe_3_O_4_ with NH_4_OH solution. Figure 1a shows nanofibers in scanning transmission electron microscopy (STEM) dark field decorated with Fe_3_O_4_ NPs. According to the gray scale analysis in Figure 1b, the NPs are in the range of 18–29 nm and did selectively form in the PB compartment. To exclude that NPs formed in solution and simply dried next to the MCNFs during transmission electron microscopy (TEM) sample preparation, we verified the location by electron tomography (ET). A series of images was recorded at tilt angles from −60° to +50° in 3° steps from which a 3D reconstruction was calculated (Figure 1c). The reconstruction indicated a helical arrangement of the Fe_3_O_4_ on the MCNFs likely following the helical wrapping of the PB. The Fe likely coordinated to the disulfide of the cross‐linked PB and was reduced to Fe_3_O_4_ in a process reminiscent to our previous Pt double helix formation.^[^ ^61^ ^]^ In contrast to the more homogeneous Pt distribution throughout the PB compartment (Figure S2, Supporting Information), Fe_3_O_4_ formed much larger, individual NPs. The energy‐dispersive X‐ray spectroscopy (EDX) analysis in Figure 1d underlines the formation of single nucleoids. The inset displays the crystal structure of the nanosized Fe_3_O_4_ NPs (Figure S3, Supporting Information). The carbonization of the Fe_3_O_4_@MCNFs followed in situ on TEM showed the stability of the loaded MCNFs during the heating process (25–850 °C). Until 400 °C the helical arrangement of the individual is still visible (Figure S4a, Supporting Information). Then, through the higher mobility of each NP, NPs start to fuse together, the helical structure vanished, and the crystallinity of the Fe_3_O_4_ NPs increased (Figure S4, Supporting Information). Figure 1e shows an atomic force microscopy (AFM) height image of Fe_3_O_4_@MCNFs. After oxygen plasma treatment, the PS corona was removed and the Fe_3_O_4_ NPs with an average particle height of ≈26 ± 2 nm became visible.

### 2.3 Bimetallic Loading

**Figure 2:**
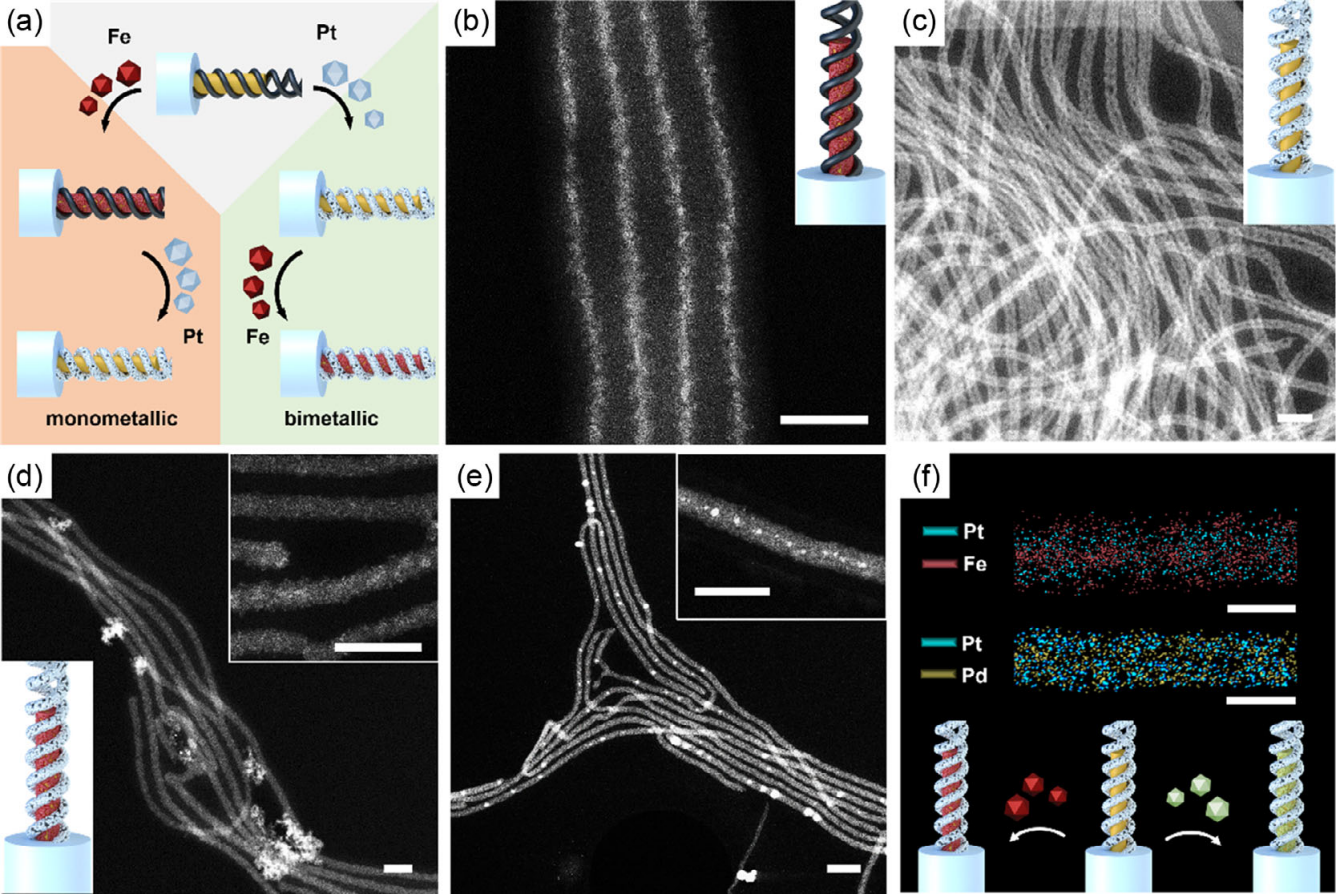
MCNFs loaded with Fe/Pt, Pt/Fe and Pt/Pd. a) Schematic of bimetallic loading. b) STEM image of Fe@MCNFs. c) STEM image of Fe/Pt@MCNFs; inset: EDX showing Pt (blue) and Fe (orange). d) STEM of Pt/Fe@MCNFs; close‐up with Fe‐loaded core (brighter) and Pt‐loaded shell. e) STEM of Pt/Pd@MCNFs; close‐up of Pd‐loaded core and Pt/Pd‐loaded shell. f) EDX of Pt/Fe@MCNF and Pt/Pd@MCNF; Pt (gray‐blue), Fe (red), and Pd (yellow); schematic clarifying location of Fe (red) or Pd (yellow). Scale bars: 100 nm.

Next, we studied the formation of bimetallic hybrids in SBMAA MCNFs using Fe/Pt as well as Pd/Pt (**Figure** **2**). We first loaded FeCl_3_ to the core followed by reduction with sodium borohydride (NaBH_4_) (Figure 2b and S5, Supporting Information). The Fe‐NPs formed in the PMAA core with a diameter of *d*_c_ ≈ 10 nm, but to some extent also in the PB domain as verified by the rough surface of the fiber with varying diameter of *d*_c+h_ ≈ 21 nm, suggesting an interaction of the Fe^3+^ with the disulfide cross‐links of the PB domain as well as the –COOH. In the second step, a Pt precursor was loaded into the MCNFs and reduced. Figure 2c shows the MCNFs after the second loading step with Pt, where the previously loaded Fe has vanished from the core, and a continuous Pt double helix becomes apparent. The EDX analysis (close‐up Figure 2c and S6, Supporting Information) confirms a complete depletion of the core and selective loading of the PB compartment. Evidently, Pt has replaced Fe by galvanic replacement (GR).^[^ ^62^, ^63^, ^64^, ^65^, ^66^ ^]^ In this case, a metal–metal surface interaction from the reduced and oxidized metal led to this replacement due to the standard reduction potential (SRP). Pt as noble metal has a high SRP (E^0^PtCl_6_^2−^/Pt^0^ = 0.744 V vs SHE) and is able to replace metals with lower SRP, e.g., Fe (E^0^Fe^3+^/Fe^0^ = –0.04 V vs SHE).^[^ ^67^, ^68^ ^]^ A larger difference in the standard potential of two participating metals causes a stronger galvanic replacement (SRP: Fe << Pt). Loading the less noble metal first therefore either results in complete replacement by the more noble metal or to bimetallic alloys.

To obtain unalloyed bimetallic MCNFs, we optimized the loading procedure and started with the more noble Pt followed by Fe or Pd (Figure 2d–f). From our previous work, we know that the Pt exclusively locates within the helical PB compartments.^[^ ^61^ ^]^ The STEM images in Figure 2d,e) confirm that the core of the MCNFs is now loaded with the second metal as well. In case of Fe, Figure 2d shows bimetallic Pt/Fe@MCNFs with an Fe‐loaded core surrounded by an inhomogeneous Pt shell. Similarly, Figure 2e shows the Pt/Pd@MCNFs with a Pd‐loaded core (brighter spots in STEM) also surrounded by a Pt shell. The overall diameter of Pt/Fe@MCNFs and Pt/Pd@MCNFs is 35 nm, which is very close to the helix diameter of the pure Pt@MCNF.^[^ ^61^ ^]^ EDX measurements provide more details about the distribution of Fe, Pd, and Pt within the MCNFs and they do not support a selective loading and thus pure metal phase of each compartment (Figure 2f). Instead, these measurements suggest that parts of the second metal accumulated within the Pt shell as well (see also Figure S7–S9, Supporting Information).

**Figure 3:**
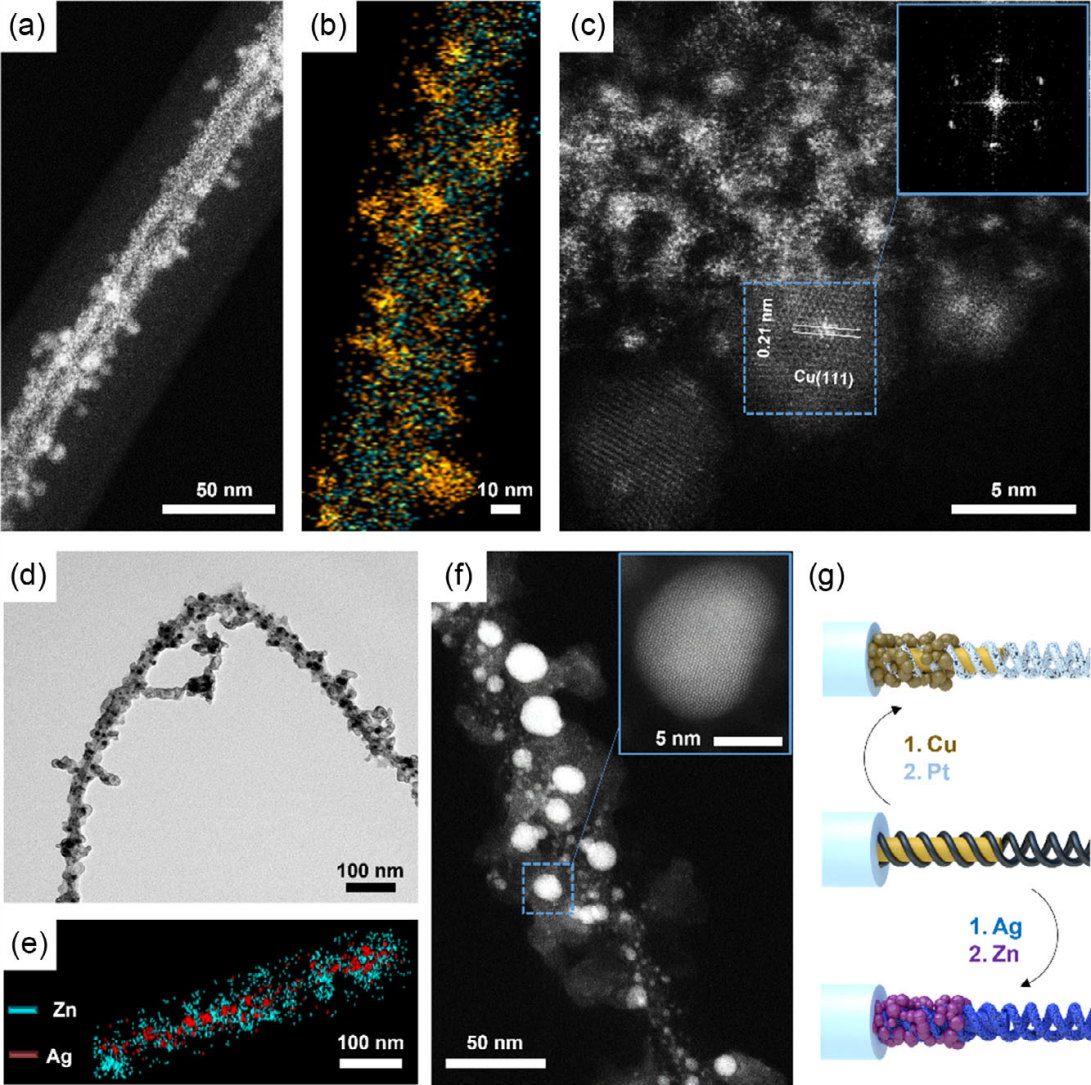
MCNFs loaded with Pt/Cu and Ag/Zn. a) STEM of Pt/Cu@MCNFs. b) Overlap of EDX spectra of Pt (blue) and Cu (orange). c) Crystal structure of Cu NP; inset FFT of Cu{111} surface. d) TEM of Ag/Zn@MCNFs. e) EDX of Zn (blue) and Ag (red) distribution. f) STEM of Ag NPs at the shell/corona interface; inset: HRSTEM of crystal lattice. g) Schematic the final hybrids.

Next, we investigated the formation of other bimetallic combinations such as Pt/Cu@MCNFs and Ag/Zn@MCNFs to verify the necessity of the optimized sequential loading procedure (**Figure** **3**). According to the SRP of Cu (E^0^Cu^2+^/Cu^0^ = 0.34 V vs SHE),^[^ ^67^, ^68^ ^]^ the Cu reduction with NaBH_4_ has to be accomplished as second loading step (SRP: Cu < Pt). Cu behaved very similar to Fe, filling MCNF core and surface pattern with a combined diameter of *d*_c+h _ ≈ 24 nm (Figure S10, Supporting Information). This suggests an interaction of the Cu^2+^ ions with the disulfide cross‐links of the PB domain as well as the –COOH in the core. After second loading with Pt, Cu is still present in the shell (Figure S11, Supporting Information). The STEM image in Figure 3a shows an overview of Pt/Cu@MCNFs with a Pt double strain decorated with crystalline Cu NPs. Compared to the formation of Cu@MCNFs in Figure S10, Supporting Information, the second loading step led to a selective interaction of the Cu ions with interface between the PB shell and PS corona, instead of the PMAA core and PB shell. The overlap of the Pt and Cu EDX measurements in Figure 3b shows the location of each metal. The PMAA core remains unloaded, while a crystallite growth on the PB domain becomes apparent (Figure S12, Supporting Information). To verify exactly the location of the Cu NPs within the MCNF, an ET was recorded. The 3D reconstruction indicated the decoration of the Pt double helix with Cu NPs, reminiscent of the Fe_3_O_4_@MCNFs. The crystal structure of the Cu NPs was recorded in the STEM image in Figure 3c. Regarding to the high‐resolution STEM and the fast Fourier transformation (FFT) of the Cu{111} surface, the difference between the Pt cluster and the hexagonal crystal structure of the Cu NPs is clearly visible.^[^ ^69^ ^]^ The interplanar atomic distance of the {111} planes is in a range of 0.21 nm, which correlates well with the *fcc* lattice parameter of 0.36 nm ($\sqrt{3}\text{/3 × 0}\text{.36 nm }\approx \text{ 0}\text{.21 nm}$). For the Ag/Zn@MCNFs, we first infiltrated the template with AgNO_3_ followed by ZnCl_2_. After reduction with ethylene glycol, the MCNFs consist of a Zn core and surface structure, which is decorated with crystalline Ag NPs (Figure 3d). In Figure S13, Supporting Information, the Zn^2+^ located within the PB domain and the PMAA core. Typically, the Ag NPs formed at the interface between the PB and PS phase (Figure S14, Supporting Information). Figure 3e displays the overlay of the EDX spectra, to underline the distribution within the MCNFs. The STEM image and close‐up in Figure 3f shows the different sizes and crystallinity of the Ag NPs. Given the polydisperse sizes of the Ag NPs, we assume a stepwise growth mechanism involving Ostwald ripening of the Ag NPs at the interface of the PB shell and PS corona.

### 2.4 Trimetallic Loading

**Figure 4:**
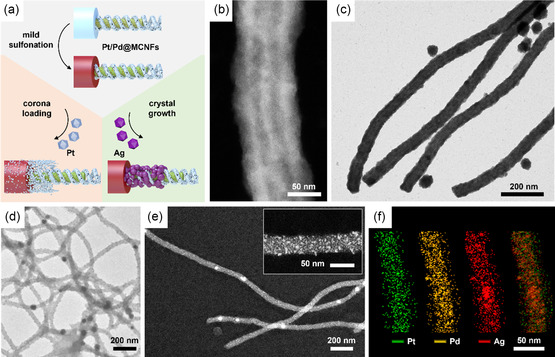
Sulfonated MCNFs loaded with two or three different metals. a) Scheme of the third loading of the MCNF corona. b) SEM and c) TEM of the Pt/Pd/Pt@MCNFs with Pt NPs on the Pt/Pd nanostructure. d,e) TEM and STEM of Pt/Pd/Ag@MCNFs with Ag NPs on the Pt/Pd nanostructure. f) EDX analysis of Pt (green), Pd (yellow), Ag (red), and overlay.

Finally, we loaded all three microdomains of the MCNFs with metals to create trimetallic MCNFs in **Figure** **4**. For that, the PS corona of Pt/Pd@MCNFs was modified to polystyrene(sulfonic acid) (PSS) by a mild and site‐specific sulfonation (Figure 4a, also S15, Supporting Information).^[^ ^70^ ^]^ Negatively charged sulfonic acid groups render the MCNFs water‐soluble and open the possibility for complexation with yet another cationic salt. The diameter of the MCNFs slightly expanded from 90 to 105 nm after sulfonation. We first used Pt^2+^ ions resulting in Pt/Pd/Pt@MCNFs (Figure 4b,c). The STEM image in Figure 4b shows the MCNFs surrounded by the Pt‐loaded PSS shell, which now has a considerably increased diameter (*d*_PSS‐Pt_ ≈ 64 nm) compared to the nonloaded one (*d*_PS‐Pt_ ≈ 35 nm, Figure S2, Supporting Information). The TEM image in Figure 4c displays the continuous Pt‐loaded PSS shell of the Pt/Pd/Pt@MCNFs that have become much stiffer as evident from the rather straight appearance as well as frequently observed fragmentation of the MCNFs (Figure S16, Supporting Information). To create trimetallic MCNFs, AgNO_3_ was added to the sulfonated Pt/Pd@MCNFs. The TEM (Figure 4d) and STEM (Figure 4e) images illustrate trimetallic Pt/Pd/Ag@MCNFs. The Ag NPs are located on the PB/PSS interface with a NP diameter of *d*_Ag_ *= *5–10 nm. Due to the electrostatic repulsion of the sulfonic acid groups, the corona expands, causing the silver ions to form a crystallization nucleus, which is easier to reach and has more space to grow. Figure 4e shows the Pt/Pd/Ag@MCNFs decorated with smaller and brighter Ag NPs. As AgNO_3_ has a higher solubility in water than in DMF (Figure S14, Supporting Information), an additional loading of the MAA core with Ag can occur in this case. The EDX analysis and the overlap of all three metal signals further supported the synthesis of the trimetallic Pt/Pd/Ag@MCNFs (Figure 4f).

## 3 Conclusion

We have reported the sol–gel formation of mono‐, bi‐, and multimetallic MCNFs using ABC triblock terpolymer morphologies as templates. The organic/inorganic hybrids resulted from selective loading of precursor salts into compartments with suitable binding sites. We synthesized various monometallic (Fe, Cu, Zn, Ag) MCNFs and tuned NP formation through GR (e.g., location of the metal NP switches from a core‐loaded nanostructure into double nanohelices). We further employed the MNCFs for the synthesis of bimetallic alloys (e.g., Pt/Fe and Pt/Pd), as well as unalloyed bimetallic nanostructures with crystalline NPs (Pt/Cu). Through postmodification of bimetallic Pt/Pd@MCNFs, the nanofibers became water‐soluble allowing the synthesis of multimetallic MCNFs with Pt/Pd/Ag in core, shell, and corona. These examples demonstrate the potential of triblock terpolymers to serve as multifunctional templates with complex shapes of the individual microdomains that can direct the growth of nanostructures and NPs. With the multicompartment properties of the template, a variety of NP compositions might become accessible with relevance for electrochemical applications or catalysis.^[^ ^71^, ^72^, ^73^ ^]^

## 4 Experimental Section

4.1

4.1.1

##### Materials

All solvents were used as received. Chloroplatinic acid hydrate (H_2_PtCl_6_, ≈38% Pt basis, Aldrich), trifluoroacetic acid (TFA, 99%, Sigma–Aldrich), sodium borohydride (NaBH_4_, 98%, Alfa Aesar), iron(III) chloride hexahydrate (FeCl_3_, 97%, Sigma–Aldrich), iron(II) chloride tetrahydrate (FeCl_2_, 99.99%, Sigma–Aldrich), Cu(II) chloride (CuCl_2_, 97%, Sigma–Aldrich), ethylene glycol (tech. grade), ammonium hydroxide solution (NH_4_OH, ≈25% NH_3_ basis, Sigma–Aldrich), silver nitrate (AgNO_3_, >99.9% metal basis, Alfa Aesar), palladium (II) chloride (PdCl_2_, 99%, Sigma–Aldrich), sulfur monochloride (S_2_Cl_2_ 98%, Aldrich), and zinc (II) chloride anhydrous (ZnCl_2_, 98%, Alfa Aesar) were used as received. The SBT triblock terpolymer was synthesized as reported previously.^[^ ^74^ ^]^

##### Hydrolysis of MCNFs

The MCNFs were synthesized as reported previously.^[^ ^61^ ^]^ After evaporation of the solvent, 20 mg MCNFs were dispersed in 20 mL DCM. Then 1 mL TFA was added to the solution to deprotect PT into PMAA, followed by stirring for 24 h at RT. For purification, the dispersion was washed twice with THF by centrifugation for 10 min at 6000 rpm. The supernatant was discarded, and the residue redispersed in 10 mL THF to set a final concentration of MCNFs in THF to 2 g L^−1^.

##### Magnetite Loading

FeCl_2_ (6.9 mg, 0.035 mmol) and FeCl_3_ (18.7 mg, 0.069 mmol) were dissolved in 3 mL DMF and added to 1 mg MCNFs dispersed in 2 mL DMF to give a final polymer concentration of 0.2 g L^−1^. The dispersion was stirred for 1 h at RT under argon atmosphere. The solution was stirred for another 30 min at 50 °C after addition of 200 μL NH_4_OH solution, followed by 1 h stirring at 80 °C under argon atmosphere. The dispersion cooled down to RT in an oil bath. For purification, the dispersion was centrifuged twice with ethanol and once in toluene. Each cycle was run for 10 min at 4000 rpm. The supernatant was discarded, and the residue redispersed in 1 mL toluene.

##### Fe/Pt Loading

The loading procedure will be exemplified on one reaction. Loading with Cu/Pt was performed accordingly (see SI). FeCl_3_ (18.7 mg, 0.069 mmol) was dissolved in 1 mL DMF and added 1 mg MCNFs dispersed in 4 mL DMF to give a polymer concentration of 0.2 g L^−1^. The dispersion was stirred for 1 h at RT under argon atmosphere. After addition of 200 μL NH_4_OH solution, the dispersion was stirred for another 30 min at 50 °C followed by 1 h at 80 °C under argon atmosphere. The dispersion cooled down to RT in an oil bath. For purification, the dispersion was centrifuged twice with ethanol and once with toluene. Each cycle was run for 10 min at 4000 rpm. The supernatant was discarded, and the residue redissolved in 2 mL DMF. For second loading, H_2_PtCl_6_ (18.6 mg, 0.045 mmol) was dissolved in 3 mL DMF, added to the Fe@MCNF dispersion to give a concentration of 0.2 g L^−1^, and stirred for 1 h at RT under argon atmosphere. For the reduction to Pt, 20 μL of ethylene glycol was added to the dispersion followed by stirring for 2 h at 80 °C under argon atmosphere and cooling to RT in an oil bath. For purification, the dispersion was centrifuged twice with ethanol and once with toluene. Each cycle was run for 10 min at 4000 rpm. The supernatant was discarded, and the residue redissolved in 1 mL toluene.

##### Pt/Fe Loading

The loading procedure will be exemplified on one reaction. Loading with Pt/Pd and Pt/Cu was performed accordingly (see SI). After evaporation of THF, 1 mg of MCNF was dispersed in 2 mL DMF. H_2_PtCl_6_ (18.6 mg, 0.045 mmol) was dissolved in 3 mL DMF and added to the solution to give a polymer concentration of 0.2 g L^−1^. The solution was stirred for 1 h at RT under argon atmosphere, followed by addition of 20 μL ethylene glycol, further stirring for 2 h at 80 °C, and cooling to RT in an oil bath. For purification, the dispersion was centrifuged twice with ethanol and once with toluene. Each cycle was run for 30 min at 6000 rpm. The supernatant was discarded, and the residue redissolved in 2 mL DMF. For second loading, FeCl_3_ (18.7 mg, 0.069 mmol) was dissolved in 3 mL DMF and added to the Pt@MCNF dispersion to give a concentration of 0.2 g L^−1^. The dispersion was stirred for 1 h at RT under argon atmosphere. After addition of 100 μL ammonium hydroxide solution, the dispersion was stirred first for 30 min at 50 °C, then 1 h at 80 °C under argon atmosphere. The dispersion cooled down to RT in an oil bath. For purification, the dispersion was washed twice with ethanol and once in toluene by centrifugation for 10 min at 4000 rpm. The supernatant was discarded, and the residue re‐dissolved in 1 mL toluene.

##### Ag/Zn Loading

For Ag loading, 1 mg of MNCFs was dispersed in 3 mL DMF and 1.7 mg AgNO_3_ (0.01 mol) dissolved in 2 mL DMF was added to give a MCNF concentration of 0.2 g L^−1^. The dispersion was stirred for1 h at RT under argon atmosphere. After adding 20 μL ethylene glycol, the dispersion was stirred for 2 h at 70 °C under argon atmosphere. After cooling down to RT the dispersion was washed one time with ethanol and once with toluene by centrifugation for 20 min at 4000 rpm. The supernatant was discarded, and the residue redissolved in 3 mL DMF. For the second loading step, 6.9 mg ZnCl_2_ (0.05 mmol) were dissolved in 2 mL DMF, added to the polymer dispersion to give a MCNF concentration of 0.2 g L^−1^. The dispersion was stirred for1 h at RT under argon‐atmosphere. After adding sodium borohydride (3.7 mg, 0.01 mmol), the dispersion was stirred for 2 h at 70 °C under argon atmosphere and then cooled to RT in an oil bath. The dispersion was washed twice with ethanol and once with toluene by centrifugation for 10 min at 4000 rpm. The supernatant was discarded, and the residue redissolved in 1 mL toluene.

##### Sulfonation of Pt/Pd@MCNFs

After evaporation of toluene, 1 mg of Pt/Pd@MCNFs was dispersed in 5 mL dichloroethane (DCE) and stirred for 15 min under an argon‐atmosphere. 3 mL of a freshly prepared acetyl sulfate solution (sulfuric acid: 1.4 mL (0.025 mol), acetic anhydride: 2.4 mL (0.025 mol)) was added slowly. After 24 h, the reaction was quenched by adding 5 mL MeOH and the DCE was removed on a rotary evaporator. The sulfonated Pt/Pd@MCNFs were centrifuged three times with water for 30 min at 4000 rpm. The supernatant was discarded, and the residue re‐dispersed in 1 mL water to set a final MCNF concentration of 1 g L^−1^.

##### Loading of Sulfonated Pt/Pd@MCNFs with Pt or Ag

To load the MCNF corona, 200 μL of the sulfonated Pt/Pd@MCNFs were dispersed in 4 mL ultrapure water. AgNO_3_ (1.7 mg, 0.01 mol) or H_2_PtCl_6_ (18.6 mg, 0.045 mmol) was dissolved in 1 mL ultrapure water and added to the dispersion to give a polymer concentration of 0.2 g L^−1^. The dispersion was stirred for 1 h at RT under argon atmosphere, followed by addition of 20 μL ethylene glycol, further stirring for 2 h at 80 °C, and then cooled down to RT in an oil bath. For purification, the dispersion was centrifuged three times with water for 20 min at 4000 rpm. The supernatant was discarded, and the residue re‐dispersed in 1 mL water.

##### Size‐Exclusion Chromatography

Size‐exclusion chromatography (SEC) was used to determine the molecular weight, weight distribution, and polydispersity of the SBT triblock terpolymer. Measurements were conducted on a 1260 Infinity (Polymer Standard Service, Mainz) instrument equipped with 3 SDV columns (pore sizes 10^3^, 10^5^, 10^6^ Å) and a refractive index detector. HPLC grade THF was used as the eluent at a flow rate of 1.0 mL·min^−1^ and 70 °C. Samples were calibrated with polystyrene standards (Polymer Standard Service, Mainz) and evaluated with the WinGPC UniChrom software.

##### NMR Spectroscopy

^1^H‐NMR measurements were done on a Bruker NEO 400 spectrometer (400 MHz, Bruker BioSpin, Rheinstetten, Germany) using CDCl_3_ as solvent.

##### TEM

TEM/STEM measurements were done on a JEOL 2200FS instrument operating at an accelerating voltage of 200 kV. Images were taken in bright or dark field mode with a 2 k × 2 k Ultra‐Scan 1000XP CCD camera (Gatan). Images were processed with Gatan software (GMS Version 2.31.734.0) or FIJI open‐source software package.^[^ ^75^ ^]^ Element specific measurements were conducted in EDX (TEM/EDX) mode.

##### TEM Tomography (ET)

For ET measurements, the TEM grid was cut in half with a razor blade to fit into the TEM tomography holder. STEM images were recorded between ± 60° in 1° steps, processed with tomviz.org software (weighted back projection),^[^ ^76^ ^]^ and visualized with Chimera software package.^[^ ^77^ ^]^

##### AFM

AFM height images were performed in air using a Bruker Dimension Icon with NanoScope V controller in PeakForce mode with SAA‐HPI‐SS cantilevers (nom. *f*_R_ = 55 kHz, *k*_C_ = 0.25 N m^−1^, Tip Radius 1 nm, Bruker). AFM samples were prepared by spin coating a small drop of solution (1 g L^−1^ in toluene) of cross‐linked, hydrolyzed, and magnetite‐loaded cylinders on a freshly cleaned (ethanol *p.a.*) piece of silicon wafer (10 × 10 mm^2^) with a home build spin‐coater at 3200 rpm for 5 s. Images were processed with Bruker's NanoScopeAnalysis software (version 1.9).

##### Scanning Electron Microscopy

Scanning electron microscopy (SEM) measurements were performed on a cryo‐field emission SEM equipped with in lens‐, chamber‐, and energy‐selective detectors for 16 Bit image series acquisition with up to 40.000 × 50.000 pixel resolution. Samples for SEM measurements were drop cast by adding a few milliliters of the aqueous sample on a silicon waver. The sample was not sputtered and imaged directly.

## Conflict of Interest

The authors declare no conflict of interest.

## Supporting information

Supplementary Material

## Data Availability

The data that support the findings of this study are available from the corresponding author upon reasonable request.
