# Supplementary material for: Multicompartment Nanostructures as Templates for Multimetallic Hybrid Materials
Source: Small Sci. 2023 Jul 21;3(9):2300071. doi: 10.1002/smsc.202300071 (PMC11935892; doi:10.1002/smsc.202300071)
Supplement: Supplementary file 1 — Supplementary Material [file SMSC-3-2300071-s001.pdf]

Supporting Information

Multicompartment Nanostructures as Templates for Multimetallic Hybrid Materials

Stefanie Tjaberings[1], Markus Heidelmann[2], Steffen Franzka[2], André H. Gröschel[1,3*]

[1] *Institute for Physical Chemistry and Center for Soft Nanoscience (SoN), University of Münster, Corrensstraße 28-30, 48149 Münster, Germany*

[2] *Interdisciplinary Center for Analytics on the Nanoscale (ICAN), University of Duisburg-Essen, Carl-Benz-Str. 199, 47057 Duisburg, Germany*

[3] *Macromolecular Chemistry and Bavarian Center for Battery Technology (BayBatt), University of Bayreuth, Weiherstraße 26, 95448 Bayreuth, Germany*

Correspondence: andre.groeschel@uni-muenster.de

Table of Contents

Supporting Videos .... Error! Bookmark not defined.

Experimental Section 2

S1: MCNFs synthesis 3

S2: Pt@MCNFs 3

S3: Fe_3O_4@MCNFs 4

S4: I*n-situ* carbonized Fe_3O_4@MCNFs 4

S5: Fe@MCNFs 5

S6: Fe/Pt@MCNFs 5

S7: Pd@MCNFs 6

S8: Pt/Fe@MCNFs 6

S9: Pt/Pd@MCNFs 7

S10: Cu@MCNFs 7

S11: Cu/Pt@MCNFs 8

S12: Pt/Cu@MCNFs 8

S13: Zn@MCNFs 9

S14: Ag@MCNFs 9

S15: Sulfonated Pt/Pd@MCNFs 10

S16: Sulfonated Pt/Pd/Pt@MCNFs 10

Supporting references 11

Experimental Section

Iron loading. For Fe loading, 1 mg of MCNFs was dispersed in 0.5 mL DMF and18.7 mg $FeCl_3$ (0.069 mmol) dissolved in 0.5 mL DMF was added to the dispersion to give a MCNF concentration of 1 g L^{-1}. The solution was stirred for1 h at rt under argon-atmosphere. After addition of 200 µL NH_4OHsolution, the solution was stirred first for 30 min at 50 °C, then 1 h at 80 °C under argon-atmosphere. The solution cooled down to rt in an oil bath. For purification, the solution was washed twice with ethanol and once in toluene by centrifugation for 15 min at 5000 rpm. The supernatant was discarded, and the residue re-dissolved in 1 mL toluene.

Copper loading. For Cu loading, of 1 mg MCNFs was dispersed in 3 mL DMF and8.5 mg $CuCl_2$ (0.063 mmol) dissolved in 2 mL DMF was added to the dispersion to give a MCNF concentration of 0.2 g L^{-1}. The solution was stirred for1 h at rt under argon-atmosphere. After addition of 4.7 mg$NaBH_4$ (0.12 mmol), the solution was stirred for 2 h at 80 °C under argon-atmosphere and then cooled to rt in an oil bath. The solution was washed twice with ethanol and once with toluene by centrifugation for 10 min at 4000 rpm. The supernatant was discarded, and the residue re-dissolved in 1 mL toluene.

Palladium loading. For Pd loading, 1 mg of MCNFs was dispersed in 3 mL DMF and18 mg $PdCl_2$ (0.1 mmol) dissolved in 2 mL DMF was added to the dispersion to give a MCNF concentration of 0.2 g·L^{-1}. The solution was stirred for1 h at rt under argon-atmosphere. After adding 20 µL ethylene glycol, the solution was stirred for 2 h at 80 °C under argon-atmosphere and then cooled to rt in an oil bath. The solution was washed twice with ethanol and once with toluene by centrifugation for 10 min at 4000 rpm. The supernatant was discarded, and the residue re-dissolved in 1 mL toluene.

Zinc loading. For Zn loading, 1 mg of MCNFs was dispersed in 3 mL DMF and6.9 mg $ZnCl_2$ (0.05 mmol) dissolved in 2 mL DMF was added to the dispersion to give a MCNF concentration of 0.2 g·L^{-1}. The solution was stirred for1 h at rt under argon-atmosphere. After adding 3.7 mg$NaBH_4$(0.01 mmol), the solution was stirred for 2 h at 70 °C under argon-atmosphere and then cooled to rt in an oil bath. The solution was washed twice with ethanol and once with toluene by centrifugation for 10 min at 4000 rpm. The supernatant was discarded, and the residue re-dissolved in 1 mL toluene.

Silver loading. For Ag loading, 1 mg of MCNFs was dispersed in 3 mL DMF and1.7 mg $AgNO_3$ (0.01 mol) dissolved in 2 mL DMF was added to the dispersion to give a MCNF concentration of 0.2 g L^{-1}. The solution was stirred for1 h at rt under argon-atmosphere. After adding 20 µL ethylene glycol, the solution was stirred for 2 h at 70 °C under argon-atmosphere. After cooling down to rt the solution was washed one time with ethanol and once with toluene by centrifugation for 20 min at 4000 rpm. The supernatant was discarded, and the residue re-dissolved in 1 mL toluene.

*Cu/Pt loading.*First Cu was loaded as described above and re-dispersed in 2 mL DMF. For Pt loading,18.6 mgH_2PtCl_6(0.045 mmol) was dissolved in 3 mL DMF and added to the Cu@MNCFs to give a concentration of 0.2 g L^{-1}. The solution was stirred for 1 h at rt under argon atmosphere. After addition of 20 µL ethylene glycol, the solution was stirred for 2 h at 80 °C under argon-atmosphere and then cooled to rt in an oil bath. For purification, the solution was washed twice with ethanol and once in toluene by centrifugation for 10 min at 4000 rpm. The supernatant was discarded, and the residue re-dissolved in 1 mL toluene.The Pt/Cu loading was done accordingly with the loading steps switched.

*Pt/Pd loading.*First Pt was loaded as described above and re-dispersed in 2 mL DMF. Pd loading was then performed as also described above.

S1: MCNFs synthesis

TEM images in Figure S1a-b show the SBT bulk film casted from $CHCl_3$ with core-shell cylinder morphology. The PT cylinders are hexagonally packed, surrounded by a continuous PB shell and embedded in a PS matrix. After cross-linking with $S_2Cl_2$the PB shell transformed into PB double helix (Figure S1b-d). Redispersion of the cross-linked bulk film resulted into individual multicompartment nanofibers (Figure S1e-f).

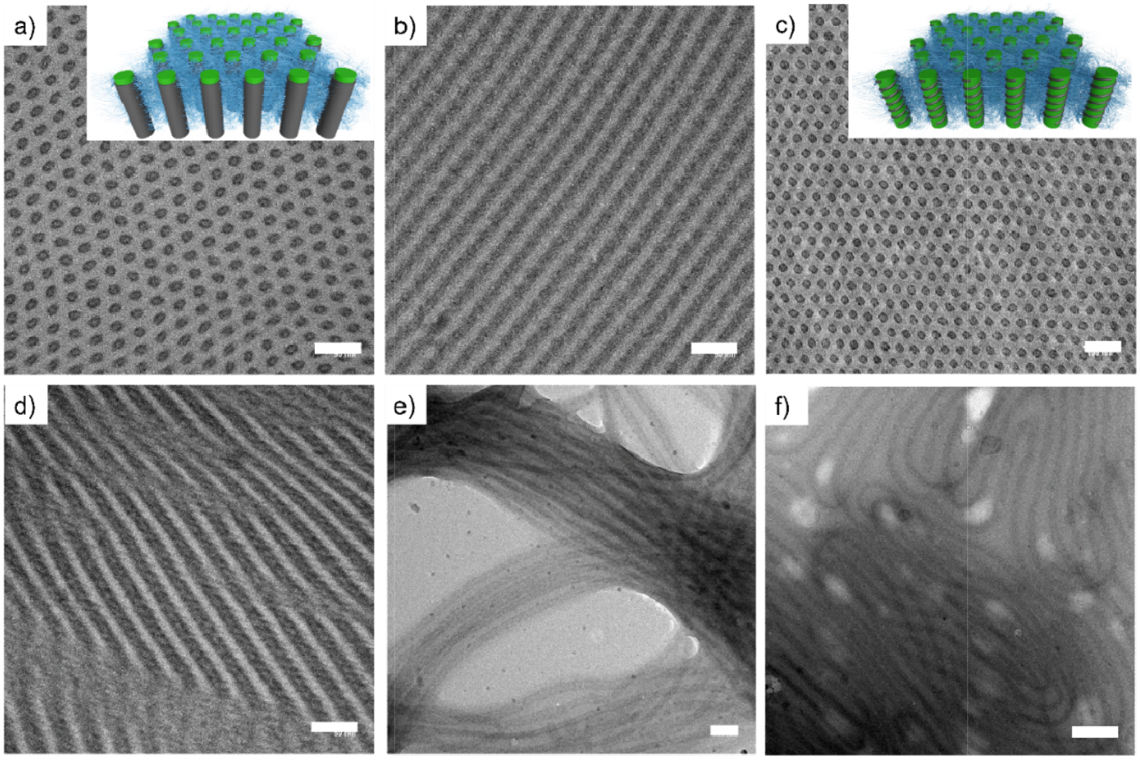

Figure S1: **TEM images of MCNF template formation.a, b)**TEM and scheme of core-shell cylinder morphology: PT core, PB shell and PS matrix. **c, d)** TEM and scheme of cross-linked helix-on-cylinder morphology. **e, f)** TEM of redispersed MCNFs in THF. Scale bars: 100 nm.

S2: Pt@MCNFs

The STEM images in Figure S2a shows a several micrometer long network out of Pt@MCNFs. Figure S2b a close-up of the Pt double helix. The Pt double helix appears bright in dark field scanning mode.

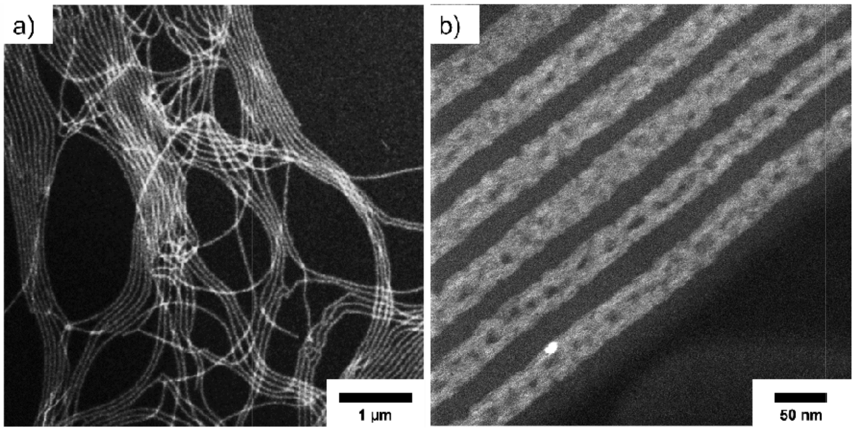

Figure S2:**STEM images of Pt double helices.a)**STEMoverview image. **b)**and close-up of Pt@MCNF.

S3: Fe_3O_4@MCNFs

The original electron image in Figure S3a shows the complementary data to the EDX image of Figure 1d. Figure S3b shows an additional STEM image of a magnetite NP and the crystal structure. The magnetite appears bright in dark field scanning mode. The EDX spectra in Figure S3b shows the characteristic $K\alpha_1$-radiation of the iron (green pixels in FigureS3b) and the characteristic $K\alpha_1$-radiation of oxygen (blue pixels in FigureS3b).

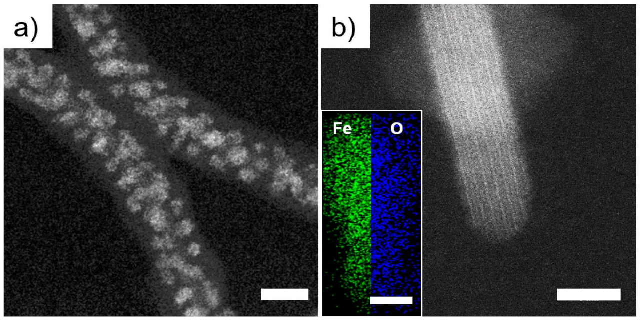

Figure S3:EDX and STEM analysis of Fe_3O_4@MCNFs.a) Original electron image of EDX analysis of Fe3O4@MCNFs. Scale bar: 50 nm.**b)** High-resolution STEM of crystalline Fe_3O_4NP (scale bar: 3 nm) and EDX measurement of crystalline NP;iron (green) and oxygen (blue). Scale bar: 2.5 nm.

S4: *In-situ* carbonized Fe_3O_4@MCNFs

The STEM images in Figure S4a shows the Fe_3O_4@MCNFs at 400 °C. The polymeric structure and helical arrangement of the magnetite NPs is still visible. Figure S4b shows the carbonized sample at rt after heat treatment up to 850 °C. The high-resolution STEM images in Figure S4 c-d show the increased crystallinity of the magnetite NPs at rt.

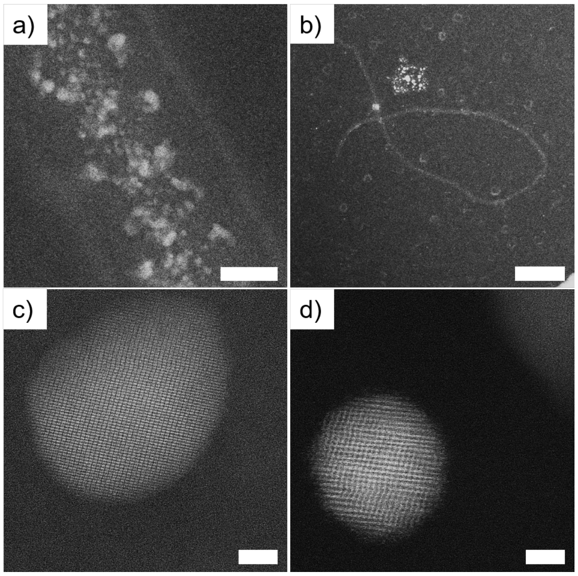

Figure S4: **STEM images of Fe_3O_4@MCNF during and after *in-situ* carbonization process.a)**Fe_3O_4@MCNFs at 400 C. Scale bar: 25nm. **b)**STEM image of the carbonized sample cooled down to rt. Scale bar: 1 µm.**c, d)**High-resolution STEM images of crystalline Fe_3O_4 NPs at rt. Scale bars: 3 nm.

S5: Fe@MCNFs

The STEM images in Figure S5a shows the location of the Fe within the MCNFs and Figure S5b a close-up of the core loaded MCNFs. The Fe core and the helix appear bright in dark field scanning mode.

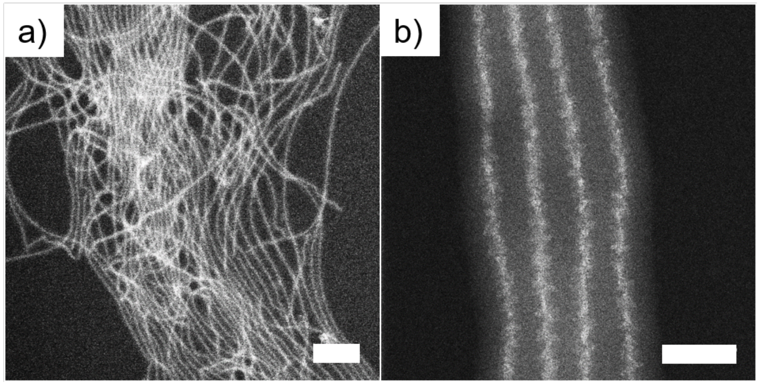

Figure S5:STEM imagesand EDX of Fe@MCNFs.a) STEM image of Fe@MCNFs. Scale bar: 1 µm.**b)**Close up of **a)**Scale bar: 100 nm.

S6: Fe/Pt@MCNFs

Figure S6 shows the original EDX spectrum of the Fe/Pt@MCNFs. Figure S6a shows the original electron image of Figure 2c. Figure S6b-c illustratesthe characteristic $M\alpha_1$-radiation of Pt (Figure S6b, blue) and $K\alpha_1$-radiation of iron (Figure S6c, green).

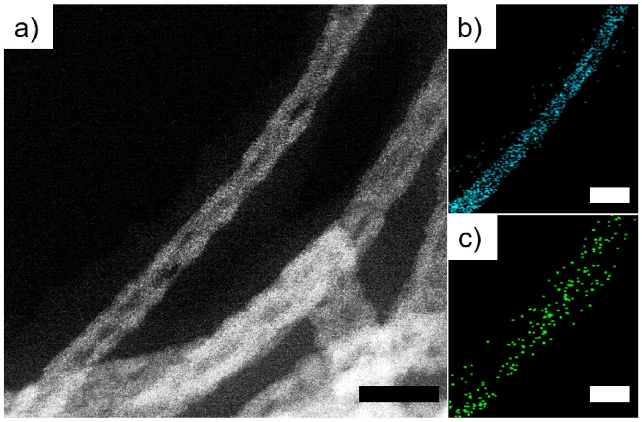

Figure S6: EDX analysis of Fe/Pt@MCNFs. **a)**Original electron imageofEDX analysis of Fe/Pt@MCNFs and the distribution of **b)**Pt (blue) and **c)**iron (green). Scale bars: 50 nm.

S7: Pd@MCNFs

The overview STEM images in Figure S7a and the close up (Figure S7b) show the length of the MCNFs and the location of the Pd within the MCNFs. Figure S7b-c demonstrate, that the PB double helix and partly the SBMAA core are loaded with Pd. The EDX spectra of the Pd double helices in Figure S7d shows the characteristic $L\alpha_1$-radiation ofPd (red).

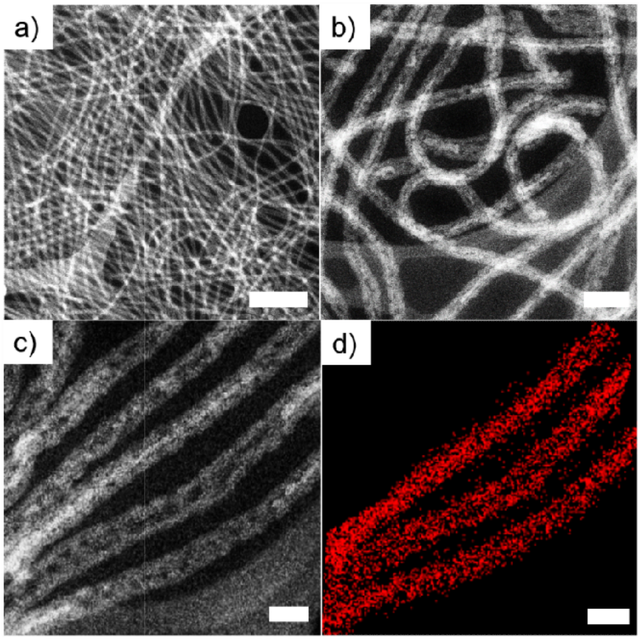

Figure S7: STEM and EDX data of single loaded Pd@MCNFs.a)STEMoverview image of Pd@MCNFs**.** Scale bar: 500 nm. **b)** Close-up of a). Scale bar: 100 nm.**c)** Original electron image of EDX analysis of Pd@MCNFs. Scale bar: 50 nm. **d)** Distribution of Pd (red) within the MCNFs. Scale bar: 50 nm.

S8: Pt/Fe@MCNFs

Figure S8a shows the original EDX analysisof the Pt/Fe@MCNFs (Figure 2f). Figure S8b illustratesthe characteristic$K\alpha_1$-radiation of iron (brown) and $M\alpha_1$-radiation of Pt (blue).

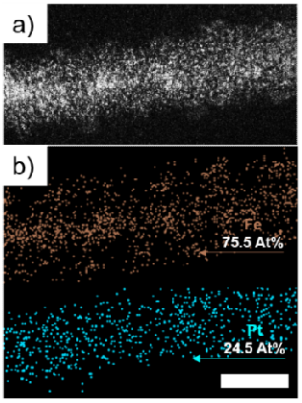

Figure S8: EDX analysis of double loaded Pt/Fe@MCNFs.a) Original electron image of EDX analysis of Pt/Fe@MCNFs. **b)** Distribution of iron (brown), Pt (blue) and composition of iron and Pt within the MCNFs in atompercent. Scale bar: 25 nm.

S9: Pt/Pd@MCNFs

Figure S9 shows the original EDX spectrum of the Pt/Pd@MCNFs (Figure 2f). Figure S9a shows the original electron image of the EDX analysis and Figure S9b demonstrates the characteristic $M\alpha_1$-radiation of Pt (blue) and $L\alpha_1$-radiation ofPd (yellow) within the MCNFs.

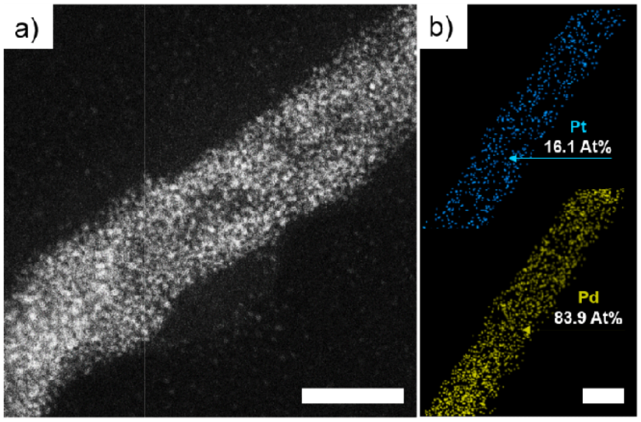

Figure S9: EDX analysis of double loaded Pt/Pd@MCNFs.a) Original electron image of EDX analysis of Pt/Pd@MCNFs. **b)** Distribution of Pt (blue) Pd (yellow), and composition of Pt and Pd within the MCNFs in atompercent. Scale bars: 25 nm.

S10: Cu@MCNFs

The STEM images in Figure S10a shows the location of the Cu within the MCNFs. Core and PB domain are loaded with Cu. The EDX of the Cu@MCNFs in Figure S10c shows the characteristic $K\alpha_1$-radiation of the Cu (yellow pixels)ofthe original inFigure S10b.

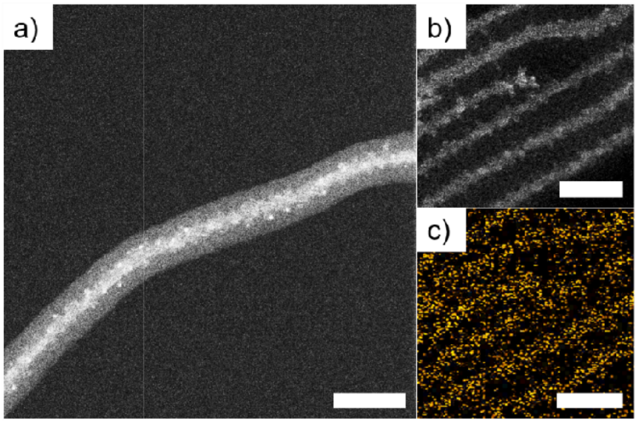

Figure S10. STEM image and EDX analysis of Cu@MCNFs. a) STEM image of Cu@MCNFs. **b)** Original electron image of EDX analysis of Cu@MCNFSs. **c)**Distribution of Cuwithin the MCNFs (yellow). Scale bars: 100 nm.

S11: Cu/Pt@MCNFs

Figure S11a shows an overview image of the micrometer long Cu/Pt@MCNFs. The close-up in Figure S11b proves that the core remains unloaded. TheEDX datais shown in Figure S11c-d, recorded from the original electron image (Figure S11c). Figure S11d illustratesthe characteristic $K\alpha_1$-radiation of Cu(yellow) and $M\alpha_1$-radiation ofPt (blue).

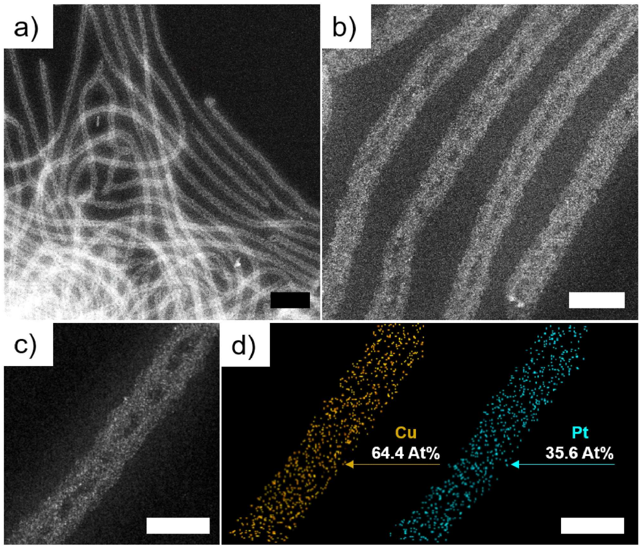

Figure S11: STEM and EDX analysis of double loaded Cu/Pt@MCNFs.a)STEMoverview image of Cu/Pt@MCNFs. Scale bar: 200 nm.**b)**Close-up of a). Scale bar: 50 nm.**c)** Original electron imageof EDX analysis of Cu/Pt@MCNFs. Scale bar: 50 nm.**d)** Distribution of Cu (yellow), Pt (blue) and composition of Cu and Ptwithin the MCNFs in atompercent. Scale bar: 50 nm.

S12: Pt/Cu@MCNFs

Figure S12a-b show the original EDX spectrum of the Pt/Cu@MCNFs (Figure 3b). Figure S12a shows the original electron image of the EDX analysis and Figure S12b demonstrates the characteristic $K\alpha_1$-radiation of Cu (yellow) and $M\alpha_1$-radiation of Pt (blue) within the

MCNFs. Additional STEM images of the crystalline Cu NP and two double loaded Cu/Pt@MCNFs are shown in Figure S12c-d.

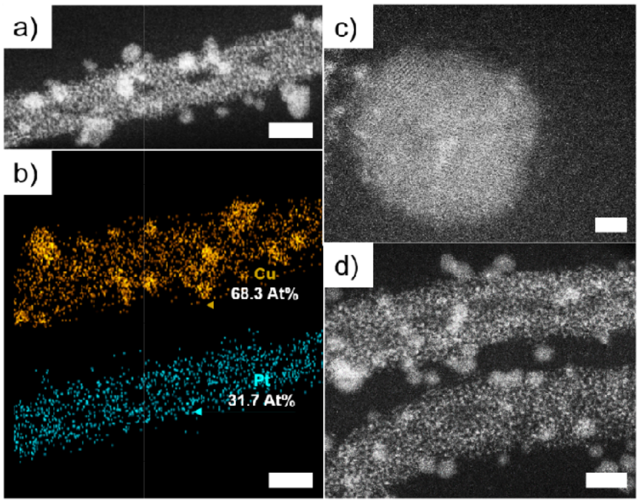

Figure S12: EDX analysis and STEM images of double loaded Pt/Pd@MCNFs.a) Original electron image of EDX analysis of Pt/Cu@MCNFs. Scale bar: 25 nm. **b)** Distribution of Cu (yellow), Pt (blue) and composition of Cu and Pt within the MCNFs in atompercent. Scale bar: 25 nm. **c)**High resolution STEM of crystalline Cu NP. Scale bar: 2 nm. **d)**STEM close-up of two Pt/Cu@MCNFs. Scale bar: 25 nm.

S13: Zn@MCNFs

Figure S13a-b show STEM images of single loaded Zn@MCNFs. The SBMAA core and the PB double helix are both loaded with zinc ions. Due to the dark field scanning mode both parts appear bright. The EDX analysis in Figure S13c shows the characteristic$K\alpha_1$-radiation of the zinc within the MCNF.

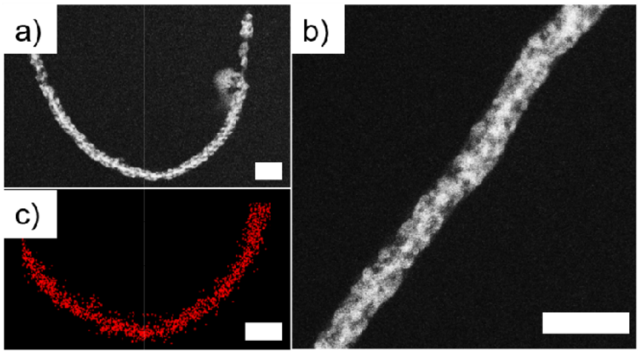

Figure S13: STEM and EDX data of single loaded Zn@MCNFs.a)STEM overview image,**b)** close-up, and**c)** EDX analysis (red: zinc). Scale bars: 100 nm.

S14: Ag@MCNFs

The STEM overview image in Figure S14a and the close up (Figure S14b) showsingle loaded Ag@MCNFs. The Agions appear bright in dark field scanning mode. The inset in Figure S14b demonstrates the EDX analysis of the silver loaded nanofibers.

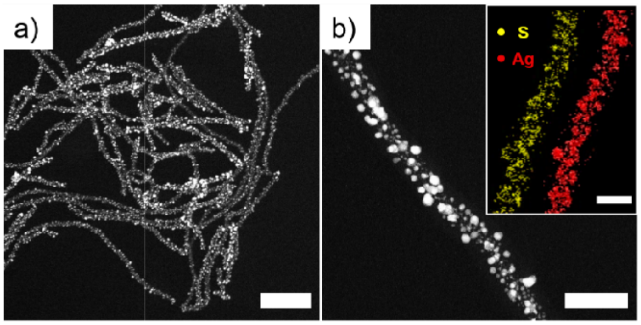

Figure S14: STEM and EDX data of single loaded Ag@MCNFs.a)STEMoverview image of Ag@MCNFs. Scale bar: 500 nm.**b)**Close-up of Ag@MCNF (scale bar: 100 nm) and EDX analysis (yellow: sulfur; red: silver). Scale bar: 100 nm.

S15: Sulfonated Pt/Pd@MCNFs

Figure S15a-b show the loaded Pt/Pd@MCNFs after the mild sulfonation process. The mild sulfonation process affected neither the structure of the bimetallic double helix nor the length of the cylinders. The EDX spectrum in Figure S15c shows the $M\alpha 1$-radiation of Pt (green) and $L\alpha_1$-radiation ofPd (red) within the MCNFs.

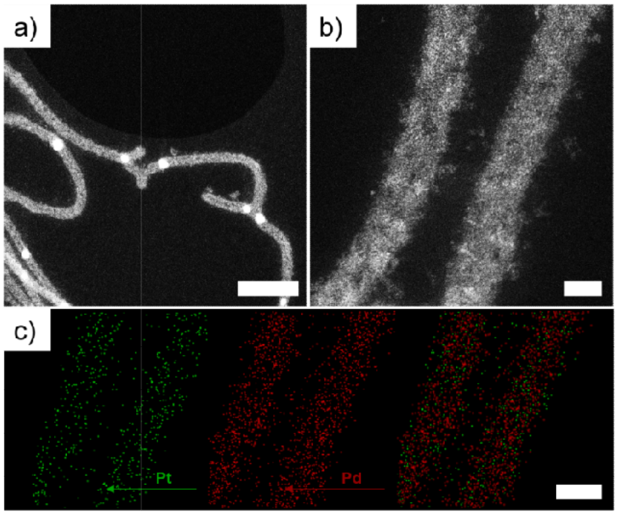

Figure S15: STEM images and EDX analysis of sulfonated Pt/Pd@MCNFs.a) STEM overview of sulfonated Pt/Pd@MCNFs. Scale bar: 200 nm. **b)** Close-up of (a). Scale bar: 25 nm. **c)**Distribution of Pt (green) and Pd (red) and EDX overlay. Scale bar: 50 nm.

S16: Sulfonated Pt/Pd/Pt@MCNFs

Figure S16 shows additional TEM, EDX and SEM images to underline the successful loading of the sulfonated PSS corona. In Figure S16a-b the previously micrometer-long MCNFs disintegrate into smaller fragments after the third loading step. This proves the increased stiffness and the higher metal content within the MCNFs.The EDX analysis in Figure S16cshows the increased amount of Pt within the nanofiber compared to **Error! Reference source not found.**f and to the Pd amount.

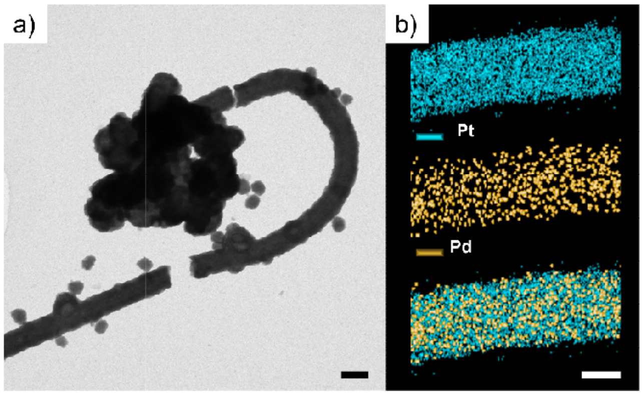

Figure S16: SEM, TEM and EDX analysis of sulfonated Pt/Pd/Pt@MCNFs.a) TEM overview of sulfonated Pt/Pd/Pt@MCNFs. **b)**EDX analysis and overlap showing Pt/Pd distribution within the MCNF. a) Scale bar: a) 100 nm and b) 50nm.

Supporting references

[1] T. I. Löbling, P. Hiekkataipale, A. Hanisch, F. Bennet, H. Schmalz, O. Ikkala, A. H. Gröschel, A. H. E. Müller, *Polymer.***2015**, *72*, 479–489.

[2] S. Tjaberings, M. Heidelmann, A. Tjaberings, A. Steinhaus, S. Franzka, B. Walkenfort, A. H. Gröschel, *ACS Appl. Mater. Interfaces***2020**, *12*, 39586–39594.

[3] J. Schindelin, I. Arganda-Carreras, E. Frise, V. Kaynig, M. Longair, T. Pietzsch, S. Preibisch, C. Rueden, S. Saalfeld, B. Schmid, et al., *Nat. Methods***2012**, *9*, 676–682.

[4] B. D. A. Levin, Y. Jiang, E. Padgett, S. Waldon, C. Quammen, C. Harris, U. Ayachit, M. Hanwell, P. Ercius, D. A. Muller, et al., *Micros. Today***2018**, *26*, 12–17.

[5] E. F. Pettersen, T. D. Goddard, C. C. Huang, G. S. Couch, D. M. Greenblatt, E. C. Meng, T. E. Ferrin, *J. Comput. Chem.***2004**, *25*, 1605–1612.
